# Development and evaluation of a symposium model for building physician-scientist skills, connections, and persistence

**DOI:** 10.1172/jci.insight.191555

**Published:** 2025-05-13

**Authors:** Kevin F. Dowling, Shohini K. Ghosh-Choudhary, Neil Carleton, Kathleen Prigg, Richard A. Steinman

**Affiliations:** 1 Medical Scientist Training Program,; 2 Department of Psychiatry, and; 3 Department of Medicine, University of Pittsburgh School of Medicine, Pittsburgh, Pennsylvania, USA.

## Abstract

High rates of physician-scientist attrition from the investigative workforce remain a significant problem despite the development of dedicated programs and initiatives designed to address the unique challenges faced by physician-scientists. However, many of these efforts are restricted to single career stages of physician-scientist training or to a single medical specialty, which may limit opportunities for beneficial vertical and horizontal mentorship regarding overcoming common career obstacles. Here, we outline the development of a physician-scientist symposium to break down silos and enable productive interactions between physician-scientists across career/training stages, academic and scientific disciplines, and medical specialties. Participants were (a) mixed in small-group problem-based discussions, (b) participated in a cross-specialty keynote panel on overcoming barriers in a physician-scientist career, and (c) took part in skill-building workshops. Attendees indicated that they fostered new connections, developed new skills to overcome career challenges, and increased their commitment to persevering in a career as a physician-scientist. Positive evaluations were not dependent on attendee career/training stage or gender. We suggest these elements of the symposium curriculum may be easily adapted for inclusion in a wide variety of physician-scientist training formats.

## Abstract 2

The manuscript presents a novel model for symposia to bolster persistence in physician scientist careers by grouping medical students through senior physician scientists to collectively discuss strategies to overcome career obstacles using case-based prompts.

## Introduction

Physician-scientists face a career of rewards and challenges. However, in too many cases the challenges are paramount, leading to voluntary or involuntary movement of talented and well-trained physician-scientists out of the investigative workforce (1). This attrition of physician-scientists conducting research has disproportionately affected women (2–4), but many systemic challenges that preclude persistence in a career as a physician-scientist are ubiquitous to all in this career path.

To date, activities that support physician-scientist career development at the predoctoral, residency/postgraduate, and early career level seek to promote career persistence by equipping individuals with the skills and knowledge needed to navigate key career hurdles. These hurdles are often common to all physician-scientists, regardless of their choice of medical specialty or academic discipline, and include finding optimal mentors at different career stages, identifying a scientific niche, acquiring and sustaining funding, building a scientific reputation, and balancing the demands of clinical practice and research. To overcome this array of challenges, budding physician-scientists need to build both horizontal support (i.e., near-peer mentoring) as well as vertical support (i.e., senior mentors and sponsors). Among the resources they can draw from, institutions and some academic societies offer formal mentorship programs to assist physician-scientists with career development and/or early-stage grant submissions. An additional challenge for physician-scientists who represent the minority of trainees and faculty in their institution is professional isolation (5). This isolation may be ameliorated to a degree by dedicated conferences for physician-scientists that foster greater connections with peers (6), including those at other institutions. However, it remains to be seen whether these initiatives have a durable effect on physician-scientist persistence.

A notable limitation of these robust efforts is that they are often siloed by training stage (e.g., confined to those in predoctoral medical scientist training programs [MSTPs], residency/fellowship PSTPs, or to early-career faculty, such as those within 5 years of their terminal degree) or are limited by academic discipline or medical specialty. Whereas peer and near-peer groups can be a source of advice and support when contending with career-stage specific or proximal challenges, the perspective they provide may be inadequate for long-term strategic planning and professional identity formation. Moreover, discipline-restricted discussions may miss practical advice in dealing with systemic challenges, strategies for productive engagement with outside partners, and innovative approaches to common career problems experienced by nearly all physician-scientists.

To address some of these limitations, we held a uniquely structured day-long Physician Scientist Symposium in June 2023 to specifically engage physician-scientists across different training and career stages, academic disciplines, and medical specialties in (a) cross-stage case-based career problem–solving activities, (b) directed skill-building workshops tailored to be of interest to physician-scientists at a range different career stages, and (c) a panel on practical strategies to overcome challenges facing individuals balancing research, clinical care, and other life roles. Participants ranged from first-year medical students enrolled in a variety of physician-scientist training programs through senior physician-scientists in academia, government, and industry. To our knowledge, this sort of physician-scientist training symposium, which presents content engaging constituencies across stage and specialty silos, has not been previously reported. We detail the development of this symposium training model, implementation of the symposium curriculum, and evaluation, which demonstrates the benefit and perceived value of this training model. We highlight how the structure and content of this symposium supports skill-, knowledge-, and network-building to promote career persistence for physician-scientists of all stages. We further highlight the features of this symposium model that could be useful in other interactive physician-scientist training or development training formats.

## Results

### Respondent characteristics.

Symposium registrants (*n* = 258) were predominately current students (*n* = 136; 53%) enrolled in either the University of Pittsburgh’s MSTP (MD/PhD) or one of several additional medical student physician-scientist training programs (unless otherwise stated, the abbreviation PSTP refers to the medical student physician-scientist training program at the University of Pittsburgh and not other postgraduate PSTPs). Student attendees reflected preclinical (*n* = 47; 35%), research phase (*n* = 73; 54%), and clinical (*n* = 16; 12%) trainees. Nonstudent registrants were resident physicians (*n* = 31; 12%), fellows (*n* = 15; 6%), faculty (any academic rank; *n* = 67; 26%), individuals who held primary appointments in industry or startups (*n* = 5; 2%), and others (defined as program officers and MD/PhD physicians currently in private practice; *n* = 4, 2%). Of registrants attending the symposium, 173 (67% of 258 registrants) completed a postsymposium survey. Registrant occupation/training categories (i.e., faculty, resident or fellow, current student, industry, or other) did not significantly differ from survey respondents (Fisher’s exact test, *P* = 0.054). Survey respondents predominately self-identified as female (female, *n* = 97, 57%; male, *n* = 67, 39%; other/not disclosed: *n* = 9; 5%). Self-identified gender did not significantly differ across occupation/training categories [χ**^2^**(8) = 3.75, *P* = 0.88] or between respondents who both facilitated/led and attended or only attended symposia panels, discussion tables, and/or workshops [χ**^2^**(2) = 1.64, *P* = 0.44].

Due to the low number of individuals who elected to not identify their gender or who identified outside of the gender binary, modeling of self-identified gender was restricted to the largest 2 self-identified groups: female and male. Similarly, analysis of occupation/training categories was restricted to the 3 largest groups, which corresponded to academic training and career stages: faculty, residents or fellows, and current students. To protect the anonymity of survey responses, training year was not collected as part of the survey; thus, differences in survey responses could not be analyzed among student subgroups. As retention of the physician-scientist workforce has been shown to differ as a function of gender (2–4), particularly at later career stages (4), a key outcome was whether any of the symposium components were rated differently as a function of these gender or career/training categories. Accordingly, statistical models were constructed to test for these effects, with gender and career/training stage categories modeled as described (see Methods).

### Perceived overall value of the physician-scientist symposium.

**Figure 1:**
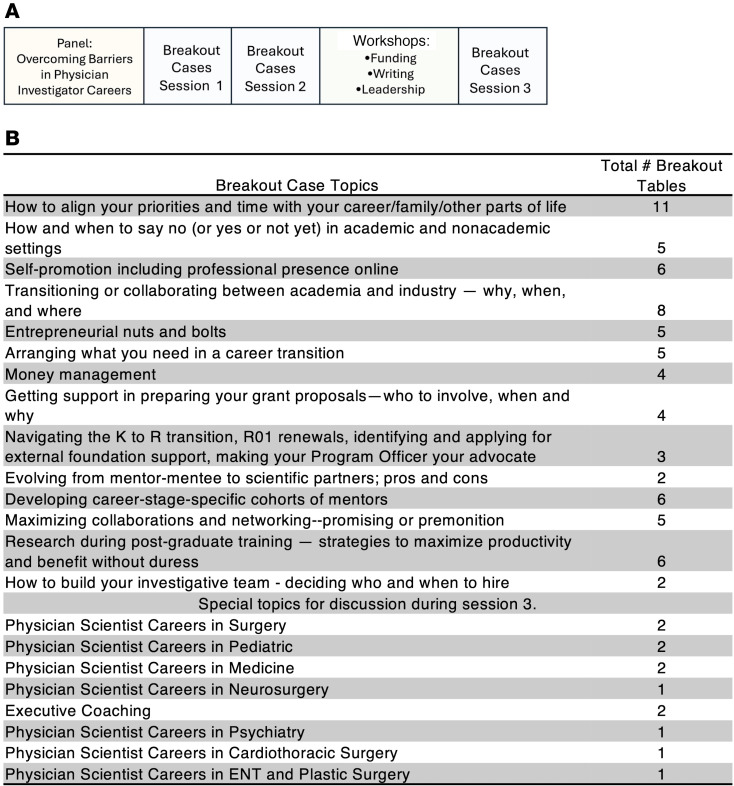
Structure of physician-scientist symposium. (**A**) Overview of the physician-scientist symposium schedule. Listed components followed a welcome/overview of symposium objectives. Each component was 1 hour in length, with consecutive components separated by a transition period. (**B**) Small-group case-based discussion topics and the number of breakout tables devoted to each case topic over the course of the 3 breakout case sessions in the symposium.

**Figure 2:**
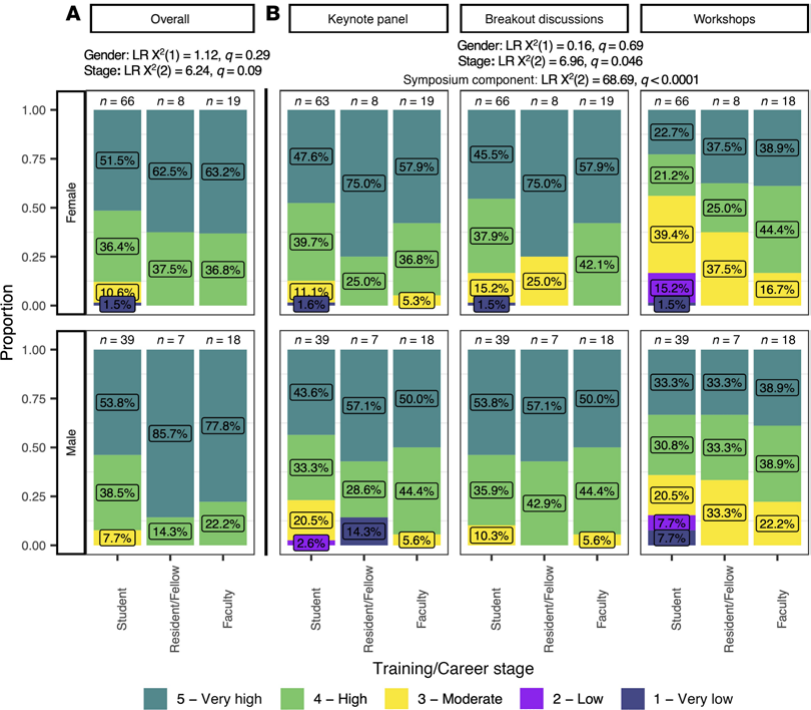
Perception of the global value of the physician-scientist symposium. (**A**) Perceived value of the symposium overall to survey respondents as a function of training or career stage (Stage, *x* axis) and for self-reported gender (rows). (**B**) Perceived value of the symposium components (columns) to survey respondents as a function of training or career stage and for self-reported gender (rows). Likelihood ratio (LR) χ**^2^** test statistics and *q* values for each predictor in (**A**) proportional odds logistic regression analysis or (**B**) cumulative logit-linked mixed-effects models are listed, separated by offsets between graphs. Results are reported as the proportion of Likert scale responses (*y* axis) in each training or career stage category (*x* axis). For each subcolumn, proportions reported as percentages are provided as inset labels for each Likert response.

The symposium structure is shown in Figure 1A and breakout table topics are shown in Figure 1B; case studies used in breakout tables are provided in the supplement materials (Supplemental Appendix; supplemental material available online with this article; https://doi.org/10.1172/jci.insight.191555DS1). Response to the symposium was very positive. On a 1 (very low) to 5 (very high) Likert rating scale, a majority (93.3%) of respondents reported that the symposium was overall of very high (*n* = 92, 58.9%) or high (*n* = 54, 34.4%) value. Overall value ratings did not significantly differ by gender [likelihood ratio (LR) χ**^2^**(1) = 1.12, *q* = 0.36] or by career/training stage [LR χ**^2^**(2) = 6.24, *q* = 0.07; Figure 2A].

When asked to assess the value of specific components of the symposium (Figure 2B), respondents’ value ratings did not significantly differ by gender [LR χ**^2^**(1) = 0.16, *q* = 0.69], but differed by career/training stage [LR χ**^2^**(2) = 6.96, *q* = 0.046] and by symposium component (LR χ**^2^** = 68.69, *q* < 0.0001). With respect to career/training stage, faculty (mean [M] = 4.4, SD = 0.6) rated all symposium components slightly higher than students (M = 4.0, SD = 0.99, *P*_Šidák_ = 0.049), overall. However, all respondents (regardless of career/training stage or gender) rated the keynote panel (M = 4.3, SD = 0.8) and breakout table discussions (M = 4.4, SD = 0.8) as of similar value (*p*_Šidák_ = 0.82), and both components were appraised to be of significantly higher value than workshops (M = 3.8, SD = 1.1, both *P*_Šidák_ < 0.0001).

### Perceived utility of the physician-scientist symposium for networking, skill building, and strategic planning.

**Figure 3:**
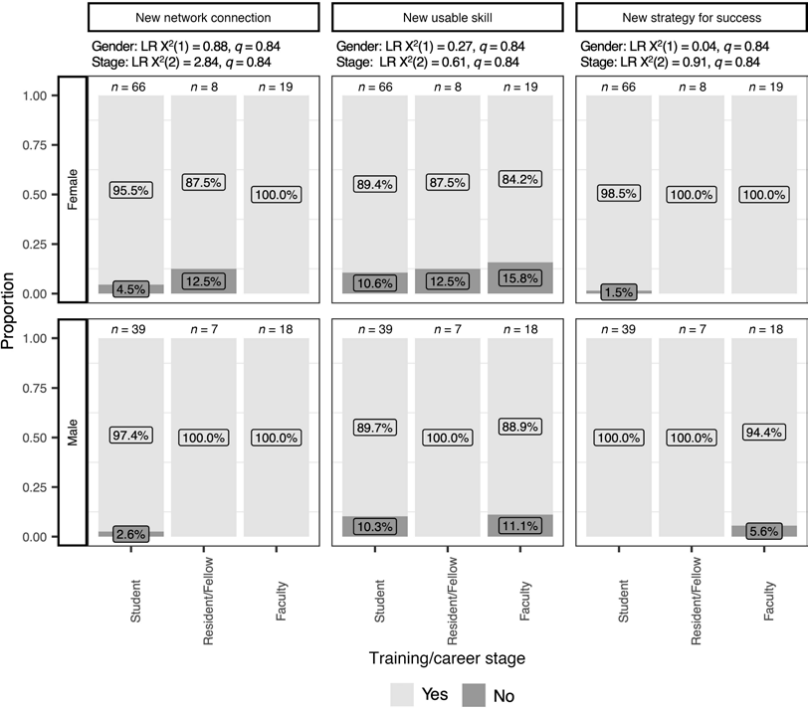
Perceived utility of symposium with respect to networking, skill building, and strategic planning. Perceived utility was evaluated as a binary outcome (yes/no). The survey items corresponding to the headers in the figure were as follows: New network connection, “the symposium introduced me to another person who could be a resource for me”; New usable skill, “the symposium provided me with at least one usable skill”; and New strategy for success, “the symposium provided me with at least one strategy that would support my success as a physician scientist.” Results are reported as the proportion of individuals responding “yes” or “no” (*y* axis) in each training or career stage category (Stage, *x* axis, subcolumns) and in each major category of self-identified gender (Gender, rows). Likelihood ratio (LR) χ**^2^** test statistics and *q* values for each predictor in logistic regression analyses are listed for the dependent variable in each column. For each subcolumn, proportions reported as percentages are provided as inset labels for each response.

Most respondents reported meeting another individual at the symposium who could be a useful resource to them (96.8%), obtaining at least one usable skill relevant to their physician-scientist careers (89.2%), or learning at least one strategy that would support their success as physician-scientists (98.7%). Whether the symposium was perceived to promote connections, provide skills, or strategies relevant to a physician-scientist career did not differ based on respondent gender [LR χ**^2^**(1) = 0.88, *q* = 0.84] or career/training stage [LR χ**^2^**(2) = 2.84, *q* = 0.84; Figure 3].

### Perceived effect of the physician-scientist symposium on commitment to career as a physician-scientist, knowledge of careers outside academia, and confidence in overcoming professional barriers.

**Figure 4:**
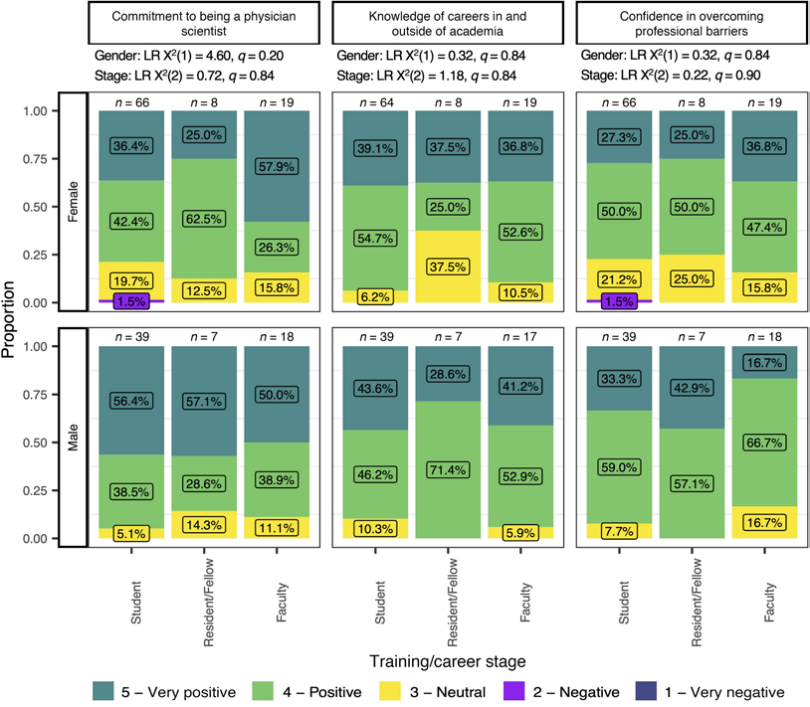
Perceived effect of the symposium overall on commitment to a career as a physician-scientist, knowledge of different careers in/outside of academia, or confidence in overcoming professional barriers. Results are reported as the proportion of Likert scale responses (*y* axis) in each training or career stage category (Stage, *x* axis). Likelihood ratio (LR) χ**^2^** test statistics and *q* values for each predictor in proportional odds logistic regression analysis are provided for the dependent variable listed in each column. Results of post hoc tests for significant predictors adjusted for multiple comparisons are reported for each row. For each subcolumn, proportions reported as percentages are provided as inset labels for each Likert response.

A primary motivation for the symposium was the need to develop interventions to facilitate persistence in physician-scientist careers. Both intrinsic factors (commitment to the career, confidence in overcoming barriers) and informational factors (knowledge of career options) can affect persistence (8, 9). We therefore asked participants to rank the effect of the symposium on these metrics. On a 1 (very negative) to 5 (very positive) Likert rating scale, on average, respondents reported the overall symposium had a very positive or positive effect on their commitment to a career as a physician-scientist (M = 4.1, SD = 0.8), knowledge of careers for physician-scientists outside of academia (M = 4.1, SD = 0.8), and confidence in overcoming professional barriers in a physician-scientist career (M = 4.1 SD = 0.8). For each of these items, ratings did not significantly differ by career/training stage [all LR χ**^2^**(2) ≤ 1.18, *q* ≥ 0.84] or gender [all LR χ**^2^**(1) ≤ 4.60, *q* ≥ 0.20; Figure 4].

In terms of symposium elements that were perceived to best bolster commitment to a physician-scientist career, the breakout tables (M = 4.3, SD = 0.8) and keynote panel (M = 4.2, SD = 0.8) were felt to be most impactful (Supplemental Figure 2A). Although, like other portions, most respondents considered the workshops to have had a very positive or positive effect on their commitment to a career as a physician-scientist, the impact rankings for the workshops were lower (M = 3.82, SD = 0.9, all *P*_Tukey_ ≤ 0.0001). Ratings of perceived impact did not differ by self-identified gender [LR χ**^2^**(1) = 3.32, *q* = 0.15] or training or career stage [LR χ**^2^**(2) = 2.69, *q* = 0.48].

With respect to respondents’ ratings of the effect of symposium components on knowledge about careers in and outside of academia (Supplemental Figure 2B), the most informative model included an interaction of gender and symposium component. In this model, there was no significant main effect of gender [LR χ**^2^**(1) < 0.00, *q* > 0.99], training or career stage [LR χ**^2^**(2) = 0.53, *q* > 0.99], or symposium component [LR χ**^2^**(2) < 0.00, *q* > 0.99] on impact ratings. The interaction of gender and symposium component was not significant after correction [LR χ**^2^**(4) = 6.83, *q* = 0.10, *P*_Uncorrected_ = 0.033] but trended toward significance, such that male respondents rated the impact of the workshops more highly than female respondents (*P*_Šidák_ = 0.018, Supplemental Figure 3). Accordingly, these results suggest that the keynote panel and interactive breakout table discussions were rated highly by respondents in both gender categories, whereas the format of the workshops was rated more highly by male respondents.

Finally, respondents’ ratings of symposium components with respect to their ability to overcome professional barriers in a career as a physician-scientist were best modeled by a model containing an interaction between symposium component and training or career stage. Neither gender [LR χ**^2^**(1) = 4.36, *q* = 0.11], symposium component [LR χ**^2^**(2) < 0.00, *q* > 0.99], nor training or career stage [LR χ**^2^**(2) < 0.00, *q* > 0.99] significantly predicted impact ratings (Supplemental Figure 2C). However, there was a significant interaction of training or career stage and symposium component [LR χ**^2^**(4) = 14.02, *q* = 0.039; Supplemental Figure 2C], such that impact ratings differed between each workshop component for students (all *P_Šidák_* < 0.0001) but not for residents/fellows or for faculty (all *P_Šidák_* ≥ 0.66). Students’ ratings were highest for breakout table discussions (M = 4.3, SD = 0.7), intermediate for the keynote panel (M = 4.0, SD = 0.8), and lowest for workshops (M = 3.8, SD = 0.9).

### Summary of qualitative textual feedback.

In addition to the quantitative survey reporting, we collected qualitative feedback from participants in free text fields. Input was obtained on what was most beneficial for the participant, on table discussions, on how participants will use information gathered, and what changes participants would make.

For faculty, the most beneficial aspect cited included interacting with individuals at various career stages (33% of comments), networking (33%), the table discussions (18%), exposure to a range of careers (15%), and information on strategies to overcome obstacles (15%). Faculty extolled specific table discussion topics, primarily those concerning work-life balance, when to say “yes/no,” and the sessions on career transitions. A representative, notable quote from a faculty member regarding engagement across career levels and medical specialties is as follows:

“I thought this event was one of the best conferences/symposiums I have attended. It was an amazing opportunity for inspiration, to meet potential collaborators and students. I’ve already had 3 students reach out to me after the conference. This was one of the only opportunities I have had to discuss research with people from surgery, psychiatry, school of public health, etc. Usually the events I attend are exclusively Dept of Medicine. I hope this can be something the University will be able to do again.”

For residents, the main benefits mentioned were the table discussions (33%) and networking (33%), followed by discussion of work-life balance (27%). An exemplary comment on the best aspect of the symposium is as follows:

“Meeting others above and below my current career level to get insight in things junior and senior researchers are encountering.”

For students, the leading benefit cited was the table discussions (37%), followed by networking (22%), information about strategies for dealing with obstacles (17%), and interacting with people at different career stages (17%). A representative, notable quote from a student regarding the table discussions is as follows:

“Balancing work/life etc. [was a favorite aspect of the symposium]. It was really great to make connections with others that have the same concerns as me and learning about what they did to overcome them and still have the career they wanted.”

In response to requests for changes to the symposium content or structure, 68% of respondents overall (57% of medical students, 90% of residents, 81% of faculty) indicated no changes or reiterated positive feedback. Two issues received more than 5% of responses. First, 13% of respondents (13% of medical students, 0% of residents, 16% of faculty) suggested decompressing the conference by spreading it over 2 days, shortening a component, or inserting social interludes. A small number or respondents wanted either an additional or one fewer table topic session. Second, 13% of respondents (18% of medical students, 0% of residents, 3% of faculty) commented negatively on the workshops. Specific issues included the content being too general or other quality concerns and the lack of a more interactive format. To address these concerns, some respondents suggested breaking up workshop content into table-based discussions.

## Discussion

In an era marked by significant attrition among physician-scientists, it is crucial for these professionals to develop robust skills to overcome common career obstacles. Additionally, they require access to a supportive, informative network that can provide guidance needed for career persistence. In keeping with this overall goal, we organized a symposium for trainees and alumni of our MSTP or other institutional physician-scientist training programs (e.g., Burroughs Wellcome Foundation [BWF] Physician-Scientist Incubator) (8) and residents/fellows or faculty who identify as physician-scientists. Individuals were brought together across academic ranks and specialties in discussion sessions focused on building practical career skills. The mixed groups worked together to frame approaches to hypothetical cases illustrating career obstacles and to share experiences. We found that this symposium format enabled practical advice to be shared horizontally between near-peers and vertically across academic and training ranks, fostered the formation of physician-scientist networks, and provided attendees with new skills to approach common challenges in a physician-scientist career.

The formation of identity as a physician-scientist is a vital element in career persistence. Sustaining that identity can be fragile in environments that cater strictly either to research or to clinical service (10). Many conferences that highlight research and clinical victories convey the value of executing the physician-scientist role successfully. Although exposure to the work of successful physician-scientists can be affirmative and motivating, consistent exposure only to highly visible and high achieving physician-scientists can leave other aspiring physician-scientists feeling isolated and more vulnerable to the unique and considerable challenges of a career as a physician-scientist that drive high rates of attrition. Therefore, our symposium specifically sought to establish a psychological space for physician-scientists at all levels to share the career obstacles they face and how they coped with them. One key conclusion arising from this event was the importance of interactive career discussions at a practical problem-solving level to attendees. Components of the symposium that are often standard features of biomedical conferences (i.e., the workshops on writing and communication, leadership, and grantsmanship) were less valued.

For problem-based small-group discussions, we intentionally recruited alumni of our institution’s various physician-scientist training programs, in order to leverage energy generated by the subset of attendees who were alumni of our MSTP/PSTP programs and who (among other facilitators) drew on their unique expertise and life-experience in strategizing how to deal with systemic and personal challenges (57% of the 51 table discussion facilitators were such program alumni). By beginning each table discussion with a hypothetical case, there was opportunity for table participants to share insights in a nonpersonal scenario prior to open-ended discussion of the table topics that generally brought personal experiences to the fore. We believe that structure helped to foster more meaningful exchanges among program attendees.

Discussion groups were distinguished by a broad range of table topics with expert facilitators, and topics were repeated throughout the day based on attendee interest. This enabled us to address issues of greatest significance to symposium attendees. The most popular topics focused on time and lifestyle management and on transitioning to or collaborating with industry. As managing competing demands on time while balancing a dual demands of a medical and scientific career is a cardinal challenge of a physician-scientist career (11), it was not surprising that, of the small-group discussion topics, sessions on how to align your priorities and time with your career, family, and other parts of life were the single most highly attended, with engagement of one-third of all symposium attendees.

The keynote panel discussion was equally popular as the table discussion groups. In the keynote panel, the 4 presenters were interdisciplinary (psychiatry, surgery, obstetrics/gynecology [OB/GYN], gastroenterology) and represented distinct career paths (industry, startup, academics) and research approaches (implementation, population health, basic/translational research, clinical research). In addition to highlighting the diversity of career paths and how panelists managed career inflection points, such as transitions from academic research to startups, the panel focused on common challenges (time limitations, leadership, system shortcomings) and how the panelists navigated these challenges (e.g., reading on sports and business psychology in order to help build coherent clinical plus research teams and dealing with “losing” grants).

Overall, at least three-quarters of respondents from every group analyzed (men, women, students, residents/fellows, faculty) stated that the symposium had a positive or very positive impact on their confidence in overcoming career barriers, their commitment to a physician-scientist career, and knowledge about physician-scientist careers in and outside of academia.

Because significant systemic barriers confront women physician-scientists, particularly (2, 12, 13), we were interested to see whether responses to the symposium differed by gender. Notably, there was no significant difference between women and men in the perceived positive impact of the symposium on their confidence in overcoming barriers, commitment to being a physician-scientist, or their knowledge of careers in and outside of academia. This could indicate the crosscutting relevance of the topics discussed, which was also reflected in the gender balance of panelists and workshop leaders (60% women) and table discussion facilitators (43% women).

Interestingly participants at all career levels, faculty, residents/fellows, and medical students, viewed the symposium highly. Both faculty and those in training indicated that the symposium deepened their commitment to their careers, broadened their toolkit for overcoming obstacles, and led to acquisition of new usable skills, strategies for success, and new networking connections. This suggests that the interactions among attendees in the symposium were not purely transactional, such as more junior attendees benefiting from the experiences of more senior attendees, but were instead of mutual benefit.

Residency and fellowship are periods when trainees must make particularly difficult decisions in order remain in the physician-scientist pipeline (14, 15). As this cohort constituted a minority of our attendees, early engagement of research residency and T32 directors in the planning and promotion of the symposium could bolster resident/fellow attendance in future multicareer level events.

A limitation of this study is that the feedback was obtained directly following the academic portion of the symposium (prior to dinner and open networking time). We cannot discern if responses were skewed by the overall tone of the event, the positive feelings arising from mentoring or being mentored in an informal faculty/trainee setting, reconnecting with peers, or by response bias. Nonetheless, we feel that the surveyed responses — (e.g., acquisition of a new usable skill, new network connection, strategies for success, commitment to persist, confidence in overcoming professional barriers) are rarely queried and should be considered as questions for most academic conferences. Finally, to discern any durable effect of participation at approximately 18 months out from the symposium, we have begun qualitative interviews of early/mid-career symposium MD/PhD physician-scientist attendees and a control group of MD/PhDs matched for graduation date.

This report offers the symposium model as an example of structured interaction between physician-scientist training program alumni and trainees and, more generally, between faculty and trainees in the context of practical advice for current and aspiring physician-scientists. The explicit focus on strategies to deal with career obstacles in an interactive format should be generalizable to other settings. Given that our measured indicators showed the cross-career stage and gender-agnostic value of this event suggests that the symposium’s format and content (see case studies and associated references in supplemental materials) could be applicable to diverse audiences and institutional settings.

## Methods

### Symposium planning.

The conference was conceived to promote career persistence and promote a durable physician-scientist identity among medical students, residents, and postgraduate physician-scientist trainees from different physician-scientist training programs in Pittsburgh, Pennsylvania, USA. A secondary goal of this conference was to facilitate connections among the alumni of Pittsburgh-based physician-scientist training programs and between alumni and current student or resident/fellow (post-graduate) trainees to cultivate vertical and peer/near-peer connections that span career stages and disciplines. Potential topics constituting common challenges or obstacles to a successful physician-scientist career were identified by the authors in consultation with an advisory committee comprising 16 alumni from the University of Pittsburgh and Carnegie Mellon University MSTP, from other University of Pittsburgh medical student scientist training programs (i.e., PSTP, ref. 7; Clinician Scientist Training Program), from the University of Pittsburgh’s BWF-supported Physician Scientist Incubator (16) program for MD/DO-only residents, and from research-track residency program directors.

The topics chosen were deemed most salient to both trainees and to alumni who would be recruited to participate in the symposium. Individualized emails were sent 10 months in advance to over 200 MSTP and PSTP alumni announcing the symposium with a description of the symposium goals and the first (of 3) requests to save the date. The invitee pool was subsequently expanded to include trainees in other BWF Physician Scientist Institutional Awardee centers, MDs newly on T32 grants, K awardees who had engaged in the University of Pittsburgh’s K-to-R training program, and MDs enrolled in any of the research-track residency options at the University of Pittsburgh Medical Center. A coordinating planning committee of 3 MSTP students, 2 staff, and the senior author was established, and the University of Pittsburgh Development Office was engaged to assist in operational implementation. Continuing Medical Education credits for participation were granted by the University of Pittsburgh, and an on-site childcare option was offered to all participants.

The symposium learning objectives as shared with participants were as follows: (a) build skills and knowledge that will support and enrich a life of inquiry, discovery, and service as a physician-scientist; (b) make connections with others that will support your development beyond the scope of the symposium; and (c) engage in mentoring with others both within and outside of your career stage.

### Symposium structure.

The symposium was conducted over the course of one full day following an optional social the evening before. The overall structure of the symposium (Figure 1A) was divided into 3 main sections. (a) a keynote panel on centered on a discussion about overcoming barriers in physician-scientist careers; (b) 3 concurrent workshops on practical career topics; and (c) a series of 3 breakout small case-based discussions on specific topics related to possible challenges in a physician-scientist career, which were interspersed throughout the day (Figure 1B). The symposium concluded with a social hour and dinner.

### Keynote panel.

The keynote panel was a facilitated discussion moderated by a member of the planning committee. The central focus of this panel was to discuss overcoming obstacles the panelists had encountered in their diverse physician-scientist careers. Panelists comprised a MD/PhD psychiatrist-scientist who left academia to establish a startup, a MD/PhD vice president at Amgen, and early career academics: a surgeon-scientist doing basic/translational research and an OB-GYN-scientist conducting clinical research. In addition to addressing questions from attendees, panelists were asked to share a brief overview of their professional career paths and fielded targeted questions from the facilitator about common barriers they had encountered while training or pursuing their professional careers and what changes they made or lessons they learned as a result of these experiences.

### Concurrent workshops.

Three workshops were run concurrently and were focused on 3 distinct topics that, in consultation with the physician-scientist advisory committee, were judged to be of practical relevance to attendees at different stages in their academic careers (for full details see Supplemental Appendix): (a) Broadening Your Research Funding Portfolio; (b) Effective Scientific Communication, Writing, and Publishing; and (c) Leadership and Building Effective Teams. During registration, attendees were given the option to rank which of these workshops they wished to attend, and almost all received their first choice. Workshop facilitators were asked to include an active component to their presentations. Each workshop was coled by content area experts, which included faculty, an NIH program officer, an executive coach, and an NIH section head.

### Breakout table discussions.

An uncommon aspect of this symposium model, relative to most other physician-scientist training initiatives, was the case-based breakout sessions that constituted the major portion of the day. Each table group included medical students, postgraduate trainees, and/or early faculty and a facilitator. Three separate rounds of case-based breakout table discussions were held throughout the day. Topics were planned and vetted by the physician-scientist symposium advisory committee. Each participant shared their preference for the 3 table breakout sessions that they wished to attend based on session topic as well as an alternate at preregistration. We ensured that each participant received at least 2 of their preferred discussion topic choices. Based on preregistration interest, multiple facilitated tables for certain topics were added.

One breakout session had career tables in addition to case-based table exercises. These provided an opportunity for networking among physician-scientists practicing in or interested in specific career paths and to enable discussion of the unique challenges associated with certain physician-scientist careers (e.g., students interested in surgeon-scientist careers and surgeon-scientists). Each attendee was assigned to 2–3 case-based topics and could attend 1 specialty-focused or executive coaching table discussion.

### Table discussion facilitators.

Table facilitators were identified by the physician-scientist advisory committee and/or solicited by the director of 3 distinct physician-scientist training initiatives at the University of Pittsburgh. Facilitators comprised 43 physician-scientists (37 academic faculty, 5 in industry, 1 at NIH), 4 postgraduate trainees, 1 medical student, and 3 professional coaches. Facilitators were selected based on known experience with the topic at hand and interest in leading discussion. Twenty-one of the faculty were alumni of our MSTP, 3 were alumni of our PSTP, and 2 were active participants in our BWF or MSTP program.

### Symposium discussion tables.

The table discussion topics and the number of assigned tables are shown in Figure 1B; topics were repeated between 1 and 3 times throughout the day based on interest for a total of 81 separate discussion tables. Additionally, topics had up to 5 concurrent facilitated tables during a session. The most popular topic, “How to align your priorities and time with your career, family, and other parts of life,” engaged 8 facilitators and was the focus of discussion at 11 tables. The table discussions were case based and included cases tailored to the experiences of students, residents, and/or faculty grouped into common career challenge-focused themes.

Each facilitator was chosen for expertise and experience in their topic area and received the case studies and the program booklet’s background reference links in advance. Facilitators were advised to use one of the topic-linked case studies as an “ice breaker” to open active discussion following participant introductions. All participants received cases and linked readings in their program booklet, sent out in advance of the meeting. There were between 2 and 8 facilitators for each of the discussion topics (average 3), depending on the level of participant interest and number of concurrent tables and sessions at which it was offered. Participants identified 4 topics of interest in advance and were matched to at least 2 of them within the 3 sessions.

### Feedback data collection.

Feedback surveys were completed by 67% (173 of 258) of participants at the event’s end. The survey respondents were asked to enter their responses on ordinal Likert or binary response scales (ordinal, 1–5; binary, yes/no) for each question (Supplemental Figure 1). Qualitative interviews of faculty participants versus matched nonparticipant controls 14–18 months after the event will be reported separately.

### Statistics.

All statistical analyses were performed using R (17). To determine whether self-identified gender and/or training status was associated with measures of perceived value of symposium components, binary logistic regression was used to analyze questions with binary (yes/no) responses. Proportional odds cumulative logistic regression analysis was used to analyze ordinal Likert scale survey responses. The R packages “MASS” and “car” were used for binary and ordinal fixed-effects models (18, 19). Because the same respondent rated the impact of multiple components of the symposium (i.e., keynote panel, small-group breakout case discussions, and workshops) for the same dependent measure (e.g., perceived value), analysis of Likert scale ratings for distinct components of the symposium were performed using proportional odds cumulative logit-linked mixed-effects models in the R package “ordinal” and “RVAideMemoire” (20, 21). For mixed-effects models, respondent ID was modeled as a random effect and self-identified gender, career/training stage, and symposium components were modeled as separate fixed effects.

For all significant main-effects models, post hoc Tukey’s honestly significant difference tests were performed to account for multiple comparisons, whereas Šidák’s test was used for post hoc testing of main-effects models to correct for comparisons across multiple variables and/or significant interactions. Multiple comparison testing was performed using the R packages “multcomp” and “emmeans” for fixed-effects and mixed-effects models, respectively (22, 23). Modeling was data-driven, with interaction terms included insofar as they improved the model’s ability to account for survey data. We compared models with and without interaction terms. Interactions were kept in the final model if the survey response data fit better when modeled as 1- or 2-way interactions (factorial) than fit as a main-effects model. Goodness of fit was indexed by the difference in Akaike’s Information Criterion (AIC) (24, 25). We report the model with the lowest AIC, unless the absolute value difference between the model with the lowest AIC and another model (ΔAIC, e.g., a factorial model and a main-effects model) was less than 2 — indicating that both models are comparable fits for the data (24, 25). In these cases, we report the results of the most parsimonious model (main effects). All model predictor *P* values were of interest and were consequently adjusted for multiple comparisons by FDR using the Benjamini-Hochberg method (26); corrected *P* values are reported in text as *q* values. *P* values of less than 0.05 were considered significant.

### Study approval.

This study was determined by the University of Pittsburgh Institutional Review Board to not be human subjects research.

### Data availability.

Values for all data points in graphs are reported in the Supporting Data Values file. Anonymized data and analysis code are available upon reasonable request from the corresponding author.

## Author contributions

All authors participated in the conceptualization and implementation of this study. RAS designed this study and acquired data. KFD performed statistical analysis. KFD, SKGC, NC, KP, and RAS contributed to the writing of this manuscript. All authors reviewed and approved submission of this manuscript. Shared co–first authorship was assigned alphabetically.

## Supplementary Material

Supplemental data

Supporting data values

## Version 1 05/13/2025

In-Press Preview

## Version 2 06/23/2025

Electronic publication
