# Supplementary material for: Development and evaluation of a symposium model for building physician-scientist skills, connections, and persistence
Source: JCI Insight. 2025 May 13;10(12):e191555. doi: 10.1172/jci.insight.191555 (PMC12220956; doi:10.1172/jci.insight.191555)
Supplement: Supplemental data [file jciinsight-10-191555-s170.pdf]

SUPPLEMENTAL MATERIAL

SUPPLEMENTAL MATERIALS: Supplemental Figures

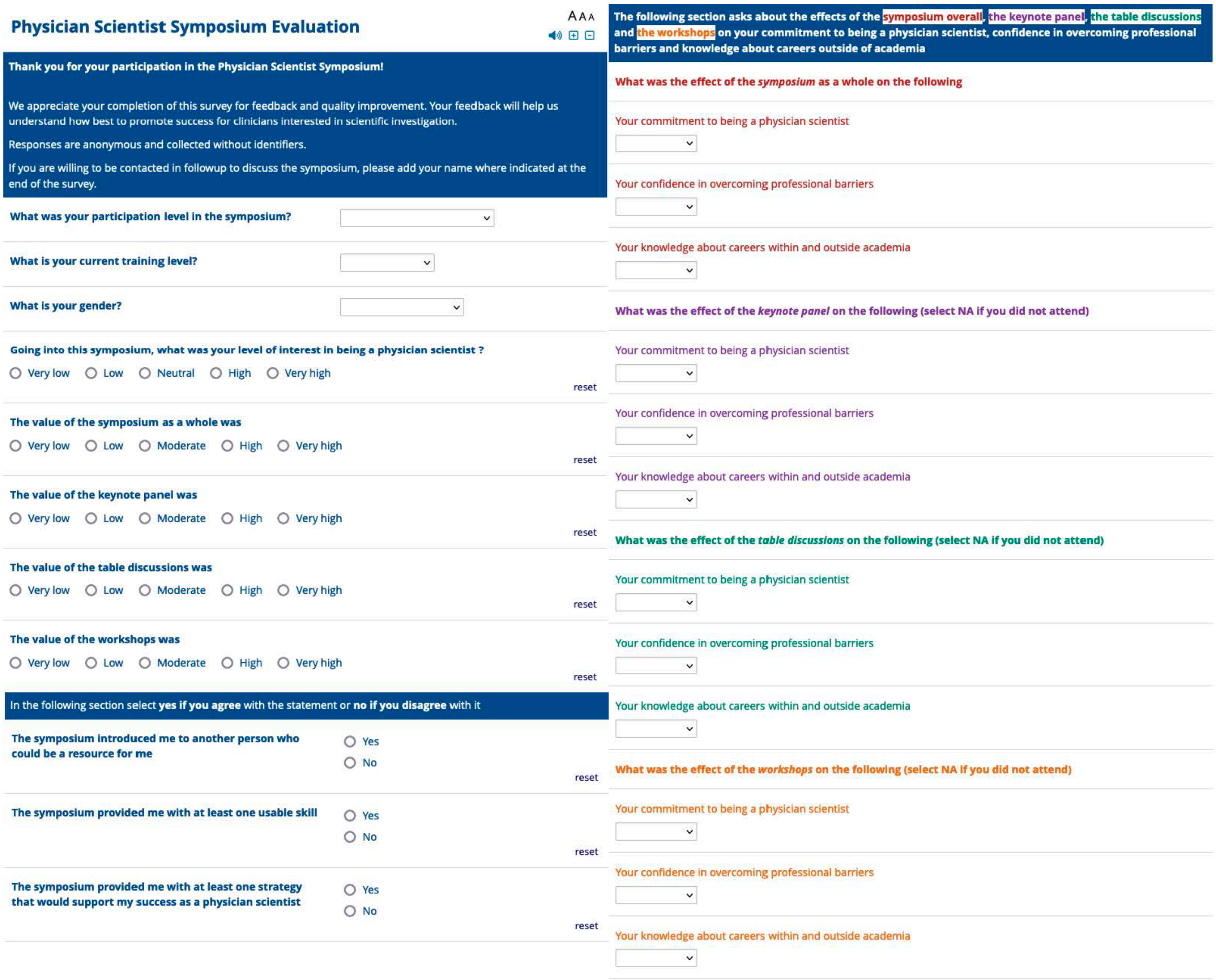

Physician Scientist Symposium Evaluation

Thank you for your participation in the Physician Scientist Symposium!

We appreciate your completion of this survey for feedback and quality improvement. Your feedback will help us understand how best to promote success for clinicians interested in scientific investigation.

Responses are anonymous and collected without identifiers.

If you are willing to be contacted in followup to discuss the symposium, please add your name where indicated at the end of the survey.

What was your participation level in the symposium?

What is your current training level?

What is your gender?

Going into this symposium, what was your level of interest in being a physician scientist ?
Very low / Low / Neutral / High / Very high — reset

The value of the symposium as a whole was
Very low / Low / Moderate / High / Very high — reset

The value of the keynote panel was
Very low / Low / Moderate / High / Very high — reset

The value of the table discussions was
Very low / Low / Moderate / High / Very high — reset

The value of the workshops was
Very low / Low / Moderate / High / Very high — reset

In the following section select **yes if you agree** with the statement or **no if you disagree** with it

The symposium introduced me to another person who could be a resource for me
Yes / No — reset

The symposium provided me with at least one usable skill
Yes / No — reset

The symposium provided me with at least one strategy that would support my success as a physician scientist
Yes / No — reset

The following section asks about the effects of the symposium overall, the keynote panel, the table discussions and the workshops on your commitment to being a physician scientist, confidence in overcoming professional barriers and knowledge about careers outside of academia

What was the effect of the *symposium* as a whole on the following

Your commitment to being a physician scientist

Your confidence in overcoming professional barriers

Your knowledge about careers within and outside academia

What was the effect of the *keynote panel* on the following (select NA if you did not attend)

Your commitment to being a physician scientist

Your confidence in overcoming professional barriers

Your knowledge about careers within and outside academia

What was the effect of the *table discussions* on the following (select NA if you did not attend)

Your commitment to being a physician scientist

Your confidence in overcoming professional barriers

Your knowledge about careers within and outside academia

What was the effect of the *workshops* on the following (select NA if you did not attend)

Your commitment to being a physician scientist

Your confidence in overcoming professional barriers

Your knowledge about careers within and outside academia

Supplemental Figure 1. Semi-quantitative survey questions symposium attendees were asked to complete following the conclusion of the academic portion of the physician-scientist symposium.

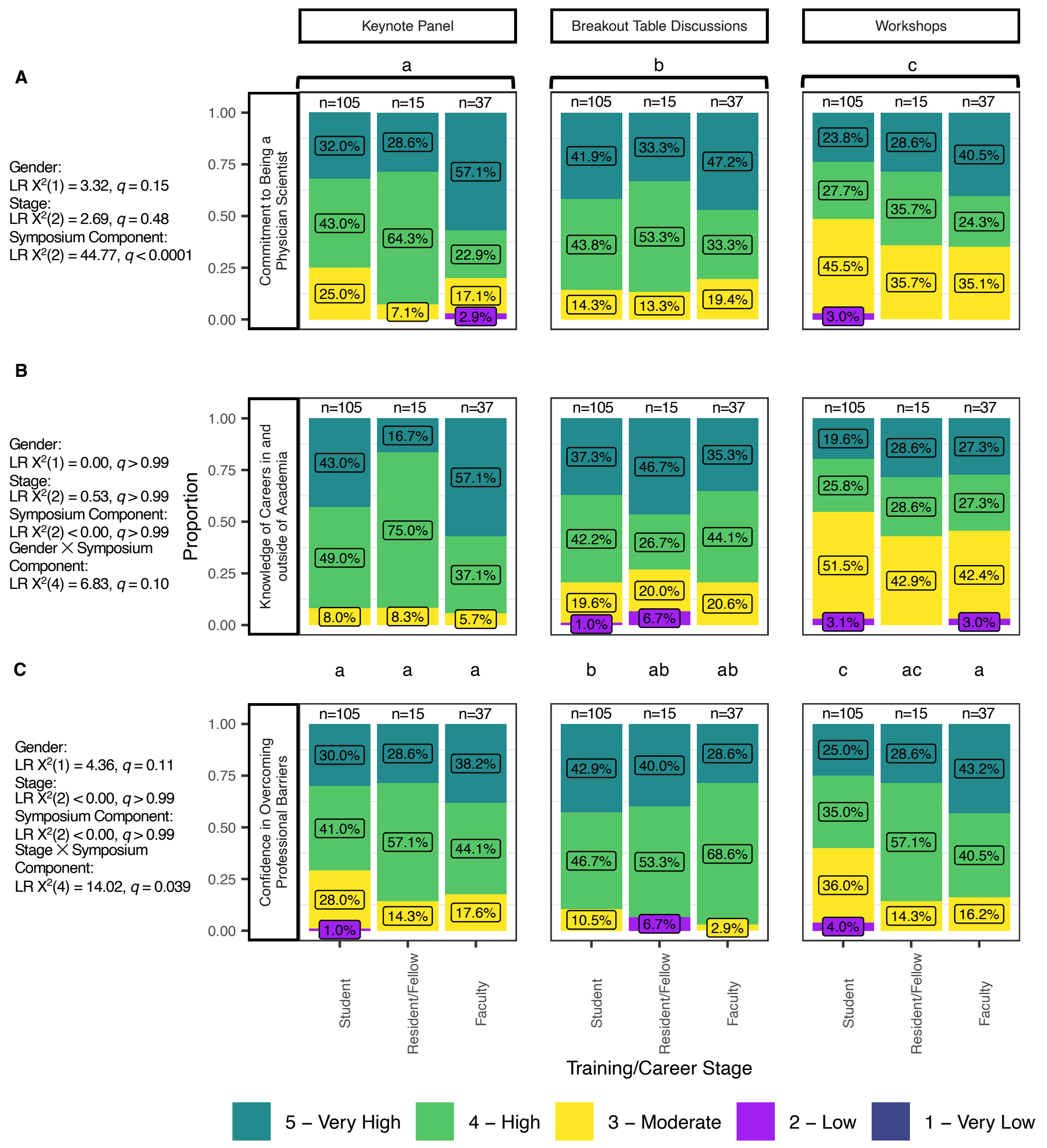

Supplemental Figure 2. Perceived effect of distinct components of the physician-scientist symposium (“Symposium Component”, columns) on **(A)** commitment to a career as a physician-scientist, **(B)** knowledge of different careers in/outside of academia, or **(C)** confidence in overcoming professional barriers (rows). Results are reported as the proportion of Likert scale responses (y-axis; colors) in each training or career stage category (“Stage”, x-axis, sub-columns). Likelihood ratio X^2 test statistics (LR X^2) and q-values for each predictor in cumulative logit-linked mixed model analysis are listed for the dependent variable in each row. Interaction test statistics are reported only when a comparison of Akaike information Criteria indicated an interaction model was a meaningfully better fit for the data. Results of post-hoc tests for significant predictors adjusted for multiple comparisons are reported for each row. Columns not sharing the same letter within row **(A)** differ from one another with respect to the effect of symposium component on the dependent variable in post-hoc tests, whereas columns (and sub-columns) within row **(C)** not sharing the same letter differ from one another as part of the significant interaction of career/training stage and symposium component on confidence in overcoming professional barriers. For each sub column, inset labels denote proportions of Likert responses for each component are reported as percentages.

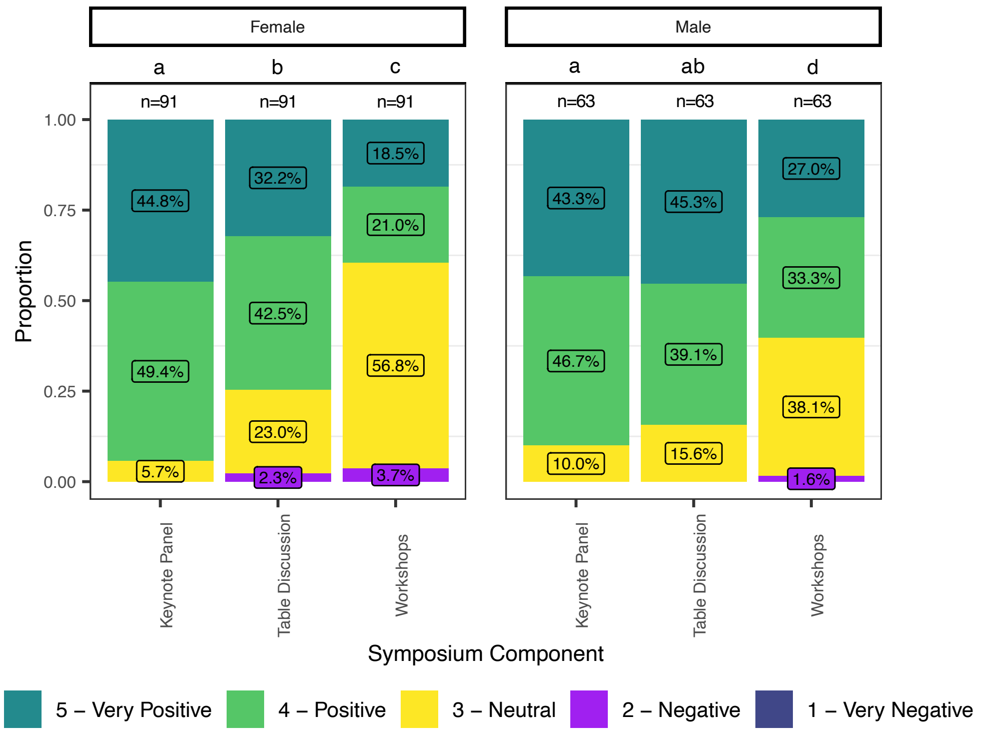

Supplemental Figure 3. Perceived effect of symposia components on career knowledge stratified by respondent self-identified gender (accompanying **Supplemental Figure 2B**) and training or career stage. Within each column (gender), letters in each sub-column (workshop component) not sharing the same letter are significantly different after correction for multiple comparisons using Sidak's test. For each sub-column (x-axis, career or training stage), proportions (as %) are provided as inset labels for each Likert response.

SUPPLEMENTAL MATERIALS: Case Studies and References from the Symposium

APPENDIX: CASE STUDIES FOR TABLE TOPIC DISCUSSIONS

TOPIC 1: How to align your priorities and time with your career/family/other parts of life

Case 1: Aisha is a tenure-track professor of medicine who is married to Jordan, a surgeon, and has two young children. She is struggling to balance her academic responsibilities with her family needs. She loves doing research and seeing patients but the time demands are overwhelming and often inflexible. Aisha has tried to set boundaries and prioritize her family, but she feels guilty when she can't attend a school play or help with homework. She cuts back on travel to conferences but is concerned about meeting tenure expectations. Moreover, her institution seems to continually add more responsibilities and required trainings and meetings. Jordan has been sharing similar complaints. How can Aisha align her priorities and time to balance her career and family obligations?

Case 2: John is a medical student pursuing a career as a physician-scientist. He faces challenges in balancing his personal needs and priorities with an ambitiousresearch project and his demanding mentor's expectations. John's self-worth is linked to his mentor's approval and being perceived as intelligent and capable by others. As a perfectionist, John obsesses over small details and ruminates on what he is not achieving. Despite working hard, his research results have been inconsistent, and he has not yet published or presented at a national conference like some of his peers. Additionally, John is concerned that he is neglecting his partner and recently missed an obligation because of his work in the lab. How can John establish realistic goals for his research while managing his personal needs and expectations of him?

Case 3: Jimmy is a surgical resident physician who wants to become like many of his idols, a surgeon-scientist. He is married with a very understanding spouse and entered his lab years

after his PGY3 years with the zeal to change clinical practice through his research. He is used to long house, significant demands from mentors and principal investigators but now has a new addition to his family. He is now finding it difficult to balance all his responsibilities and doesn't want to let his wife, new son, or his faculty mentors down. He continues to reluctantly take on more responsibilities but deep down knows he isn't giving 100% to everyone which is starting to affect his sleep, mood, family dynamics and quality of his research. How can Jimmy balance his research responsibilities, clinical responsibilities while navigating being a new dad?

- Reference 1: *Stories From Early-Career Women Physicians Who Have Left Academic Medicine: A Qualitative Study at a Single Institution*
- Reference 2: *Physician Resilience What It Means, Why It Matters, and How to Promote It*
- Reference 3: *An Integrated Career Coaching and Time-Banking System Promoting Flexibility, Wellness, and Success: A Pilot Program at Stanford University School of Medicine*

TOPIC 2: How and when to say no (or yes or not yet) in academic and nonacademic settings

Case 1: Darrell is a surgery resident who has just returned to a PGY4 clinical year after 2 years primarily spent in research. He has a published manuscript, 2 review articles, 4 co-authorships and multiple talks from his research years, but still has 2 manuscripts to wrap up. Darrell is both nervous and excited about his clinical reentry. He is on a very busy service. While operating with the senior attending from his clinical research project Darrell is asked to take over and complete a follow-up project. The day before he agreed to review a manuscript for another attending, and also received an email from a friend and colleague to recruit participants for her project on resident-medical student communication. Darrell has a reputation for excellent performance and never saying no. How and when should he add that to his vocabulary?

Case 2: Maria is a new assistant professor who is still learning to navigate the academic world. She has some early success with V-Foundation support for her small investigator-initiated clinical trial. She teaches students and housestaff and enjoys her service on the School of Medicine Admissions Committee and Department's new DEI committee. Maria loves her job but is beginning to feel overwhelmed though. But at her latest meeting with her division chief, he indicated that she should serve on the Division's Protocol Review Committee and/or the University IRB and that adding this would be more aligned with her work than her other commitments. How should she proceed?

- Reference 1: *Clinician-trialist rounds: 15. Ways to advance your career by saying 'no' – part 3: how to say 'no', nicely*
- Reference 2: *Clinician-trialist rounds : 13. Ways to advance your career by saying 'no' – part 1: why to say 'no' (nicely), and saying 'no' to email*

TOPIC 3: Self-promotion including professional presence on-line

Case 1: Justin Case, MD is terrible at self-promotion. He is about to enter the room for a critical meeting in which he has to promote himself. In a faculty interview elsewhere, Justin thought he did well but did not make the 2nd interview pool. He reflects on the phrases he had used and remembers the facial expressions of that interviewer which were hard for him to read. He doubts his ability to 'read the room.' Justin knows from his CV and previous accomplishments that he is a competitive candidate. He however struggles at switching from typed conversations, CV writing, paper writing, and transferring this into words. How can he do his best in this situation?

Case 2: Dr. Esa Payne's paper is published in a great journal. She has discovered a group of neurons in the brainstem that inhibit pain. Male scholars are far more likely than females to self-promote their high impact papers, and Esa is no exception. She tends to be conservative in

stating and disseminating her accomplishments. She looks up a male colleague's twitter postings. He highlights his papers, gives links and describes how it is "novel," "unprecedented" and "remarkable." This is not her style. Still, Esa friend prompts Esa to put her paper announcement on Twitter. Esa's tweet praises her team in helping to uncover both this inhibitory mechanism and the activator of these neurons. She is pleased to have highlighted that the paper is important without seeming arrogant in the process. Any suggestions for Esa's self-promotion?

- Reference 1: *Tweeting from the Bench: Twitter and the Physician scientist Benefits and Challenges*
- Reference 2: *Pearls of wisdom for aspiring physician-scientist residency applicants and program directors*
- Reference 3: *How to Self-Advocate for your Science and Career*

TOPIC 4: Transitioning or collaborating between academia and industry — why, when, and where

Case 1: Dr. Kim is a physician-scientist at a prestigious academic institution, where she has made significant contributions to the understanding of the pharmacology of a novel drug for the treatment of cancer. However, she has become frustrated with the slow pace of academic research and burden of writing continual grants with only rare success. She has begun to consider a move to industry, where she believes she could have a greater impact on the development of cancer therapeutics. Her main concerns about a move would be about losing the autonomy and intellectual freedom that she enjoys in academia and missing the academic environment. She also wonders if she lacks knowledge to effectively navigate a business environment. Dr. Kim reaches out to a friend who is now in pharma to ask their impression of the benefits and drawbacks of a career in academia versus industry/pharmaceuticals. What might she learn?

Case 2: You are early in your faculty career. You have provocative preliminary data in cells and mice indicating that the much-lauded drug CancerGone™ had a different target than current dogma. You showed that the drug's efficacy against multiple cancers (at least in mice) was predicted with 98% accuracy by the level of a urine metabolite of that target. You had reached out to CancerGone Pharmaceuticals with a letter of intent seeking their funding for human studies of this predictive biomarker and they agreed to review your proposal. While awaiting their decision, a colleague chides you for not filing an invention disclosure first or at least talking with the tech transfer office. You have no idea what she is talking about. She says that you can never trust industry not to steal your ideas. This strikes you as very cynical; in your opinion collaborating with CancerGone Pharmaceuticals will be much more straightforward than struggling to get funding from NIH. What are myths and facts about collaborating with industry?

- Reference 1: *Physician scientist Careers in the Biotechnology and Pharmaceutical Industries*
- Reference 2: *From academia to industry: a road more travelled*

TOPIC 5: Entrepreneurial nuts and bolts

Case 1: Dr. Marisa Borg, a clinician scientist, identified an opportunity to create a software platform that would facilitate collaboration between physicians and researchers. Despite having limited experience in entrepreneurship, she was determined to pursue her idea based on her view that poor communication between physicians and scientists slowed medical advances. She enrolled in business courses and worked with a team of advisors to develop a business plan and secure funding. Unfortunately, the project failed to gain traction and only rarely researchers and clinicians were interested in expanding their workload to explore collaborations. Also, Dr. Borg is concerned that her academic performance is suffering as she tries to manage

the unanticipated entrepreneurial demands. And so her SynapsaDoc company was dissolved. What practical steps could have bolstered the company's prospects?

<u>Case 2:</u> Jamal Reed, a medical student, brought together a team to compete in the Randall Big Idea Contest for budding entrepreneurs. They created AccuSkin: Bridging the Racial Divide in Dermatology with Technology. This was a prototype mobile app to help physicians diagnose skin conditions. Notably, it had as high an accuracy in Black patients as in Whites, a major gap in dermatology training. The team includes classmates expert in image analysis, software development, a dermatology resident, and a professor from the Katz school of business. What / who are they missing?

<u>Case 3:</u> Sally is a surgical resident in her second year in the lab which focuses on a developing a new technique of protein analysis. They've had many successes in their lab and are excited that their new technique is giving superior results to current methods. This technique uses a propriety device that her lab has worked on for the last 5 years. They are excited to present their findings at a medical device conference in Italy as well as publishing their findings in a proteomics journal. One of Sally's friends mentioned if she was worried about the proprietary technology and methods being picked up or stolen by industry or other researchers. She never thought about this, and now has reservations about when and how to present her data safely. She doesn't even know if her research is considered intellectual property. Should Sally keep on her trajectory and present her labs findings? Should she seek counsel and hold off on presenting? Is her methods or device something that should be patented and how would she even go about that?

- Reference 1: *MIT OPENCOURSEWARE, Nuts and Bolts of Business Plans*
- Reference 2: Digital startups, where to begin, https://psychnews.psychiatryonline.org/doi/10.1176/appi.pn.2023.03.3.42

TOPIC 6: Arranging what you need in a career transition

Case 1: Brad Steele is an MD/PhD who trained in Psychiatry at UCSD in the R25 research resident track and is now looking for a faculty position. He has clear goals and objectives, a plan for how to achieve them, and has overcome his natural modesty to clearly account for his substantial strengths. He received a $15K AACAP Pilot research award, submitted a Brain and Behavior Young Investigator Award application and is drafting a K award. Brad received a travel award/podium talk at Society for Neuroscience and has 2 publications. He ran his strategic plan for his research on Microbiome Contributors to Pathological Selfishness past several friends and an Assistant Professor at his institution. He has received offers to interview and give a chalk talk at several prestigious institutions. Brad has decided that to succeed he will need 70% protected time for his research and a 600 sq. ft. laboratory. He is deciding whether to request a postdoc, technician or both. How likely is Brad to procure a faculty position? Is there anything that he has overlooked? What might shape the outlook of prospective employers?

Case 2: Elissa Hasty is interviewing for residency positions. She is a trainee in the Physician scientist Training Program at University of Pittsburgh, a program with a 6-course professional development and research enrichment curriculum and 16 months of committed research. Elissa is interested in a research residency/heme-onc fellowship. She has a paper from her work on pro-tumorigenic transfer of mitochondria from fibroblasts to cancer cells. Everyone else on the interview trail has MD & PhD degrees. What details of a research pathway residency/fellowship should she focus on to ensure a great experience in this pathway and a successful transition to junior faculty? What are the questions that Elissa should ask at every interview?

- Reference 1: *Transitioning from Fellowship to a Physician Scientist Career Track*
- Reference 2: *Getting a Job, Physician Scientist Career Guide*
- Reference 3: *Getting a Job*

TOPIC 7: Money Management

<u>Case 1:</u> Dr. Sophia Martinez recently completed her fellowship in infectious diseases at a major academic medical center in Boston and is about to start as an assistant professor at the same institution. She had paid off $100,000 of her student debt by successfully competing for a NIH Loan Repayment Award during 2 years of fellowship research. She has a remaining loan debt of $70,000 with a variable interest rate of 6.8%. She is interested in purchasing a home within the next year and has been looking at properties in the area. She was interested to hear about the physician’s loan option to avoid a down payment and mortgage insurance. Her starting salary is $160,000 per year, and she will receive an institutional match of up to 5% for her retirement savings. She decides to contribute 10% of her salary to a Roth 401(k) account.

She is organized and generates a budget. The cost of living is higher in Boston than in Pittsburgh. Dr. Martinez's estimates her monthly take-home pay to be $8,500, and after reviewing her expenses, she determines that she can afford a mortgage payment of $2,500 per month. She works with a financial planner to refinance her student loans to a fixed rate of 4%, reducing her monthly payment by $100 each month. This will go towards her emergency fund. She and her partner had always discussed starting a family after her training. Even though Dr. Martinez was thorough and expert in planning the budget for her (awarded!) K99/R00 grant, she is much less sure about planning expenses for a new family. She worries that the 2-bedroom 800 square foot condo that they hope to buy will be caught up in a bidding war and will soon be too small for their family. Her department chair mentions a Saturday morning moonlighting clinic for $1500/session and she has watched a colleague do legal consulting work for $500/hr. Her partner also thinks that their 10-year-old Honda is reaching the end of it’s lifespan. What will Dr. Martinez need to consider?

Case 2: Luna Youngstar, MD, PhD is starting her own lab and is planning her budget. She needs to decide whether to buy equipment or use shared equipment; shared equipment could save her money but could be difficult to schedule and slow productivity. Although she has a generous start up package, she is conservative by nature and chooses to rely on common equipment dispersed over adjacent floors and only hires one technician to start with. Animals are very expensive to house at her institution. Her in vivo drug studies have a short timespan. She is considering purchasing animals at need versus breeding and housing costs, and decides to buy enough breeder pairs that the first generation should give enough offspring for her experiment. In terms of her grant strategy, Dr. Youngstar is deciding how much effort to spend on small grants without indirects versus large federal grants. She had received society grants as a fellow and notes that there are many of these directed at young faculty. She'll considers putting in a 2 year R21 grant to NIH next year. She mentions this plan to a friend working in a senior investigator's lab nearby. Her friend advises her that indirects will be important to cover the rent. Dr. Youngstar did not realize that she would need to pay rent for her laboratory. She also overhears her clinical colleagues talking about the $20k bonus they just received and wonders why her research contributions/potential are not equally valued within her department. What advice would you give Dr. Youngstar to help her make the best decisions for her lab's long-term success?

- Reference 1: *Systematic review of personal finance training for physicians and a proposed curriculum*
- Reference 2: *The AAMC Financial Wellness Program*

TOPIC 8: Getting support in preparing your grant proposals — who to involve, when, and why

Case 1: Gloria Gutman, MD is a young gastroenterologist-scientist who studies the gut microbiome's role in the development of inflammatory bowel disease (IBD). She plans to submit

a K23 proposal to the National Institutes of Health (NIH) to support her work. Before submitting her proposal, Dr. Gutman reached out to a program officer at the NIH to discuss her research idea and get feedback on her proposal's feasibility. The program officer provided valuable feedback, including suggestions for optimal study sections for the proposal. Dr. Gutman searched NIH reporter and found that an immunologist at her institution used to be on a study section that funded an IBD/microbiome R01. She asked for his comments on her Aims page. She included experts in gut microbiome research, biostatistics, and clinical trial design in her training plan. She worked very hard but realized that with 5 weeks to go she had not circulated the first full draft to a key individual for comment. She was also running behind on budget and justification that she left until last and on gathering letters of support and recommendation. It would be tight but she thinks she can manage all of that. Should she submit?

Case 2: Mikhail Voronin is working on a F32 training grant during fellowship. He has been working on a mechanism through which white blood cells accelerate clotting in DIC. As required by NIH, it is original and largely independent of his sponsors funded research projects. He is confident about the science excerpt for a subaim using -omics for which he has no background. He thinks that his “Applicants Background and Goals for Fellowship Training” is OK but not great and he is most nervous about the “Sponsor and Co-Sponsor Statements” that his mentor has told him to draft. He has a lot of time as he is 2 months from the submission deadline. Who and what resources and training can Mikhail pull in to make sure that the submission is as strong as possible.

- Reference 1: *How to Develop and Write a Grant Proposal*
- Reference 2: *Demystifying the NIH Grant Application Process: The Rest of the Story*
- Reference 3: *Demystifying the Logistics of the Grant Application Process*

TOPIC 9: Navigating the K to R transition, R01 renewals, identifying and applying for external foundation support, making your Program Officer your advocate

Case: Billi Livermore, MD is a physician-scientist who has been awarded a K08 grant for her research on the molecular basis of liver diseases. She has made significant progress in her research project and has published several articles in high impact journals. As her K08 grant is coming to an end, Dr. Livermore is preparing to apply for an R01 grant to continue her research as an independent investigator. She is concerned that her plan still has too much overlap with her mentor's focus and that she has not built an independent cohort of collaborators. Dr. Livermore's mentor had suggested that she attend grant writing workshops and reach out to her Program Officer at the National Institutes of Health (NIH) to discuss her research project and get guidance on the grant application process. She schedules a meeting with her Program Officer sharing her biosketch and Specific Aims. The Program Officer expresses some concern about ongoing overlap with her mentor and who notes that the mentor is senior author on all her papers.

Based on Dr. Livermore's case, what are some common challenges that physician-scientists face when transitioning from K to R awards, and what strategies can be used to overcome these challenges? How can a Program Officer serve as an advisor and guide for the grantee during the K to R transition?

- Reference 1: *K Awardee to R Advancement Training (KARAT)*
- Reference 2: *Thirty-two steps for getting your R01: advice to early career investigators*
- Reference 3: *We don't bite! Communicating with your program officer*
- Reference 4: *Tips for Communicating with Program Officers*

TOPIC 10: Evolving from mentor-mentee to scientific partners – pros and cons

Case: Dr. Carla Pillman is a clinical research fellow who has just accepted a faculty position at the same institution where she completed her fellowship. Her mentor, David Smith, MD, PhD, is an established physician-scientist in the same research field. They have a very good relationship and Carla published multiple papers on psychosocial determinants of medication adherence under her mentor's guidance. Carla wants to sustain collaborations but distinguish herself from her mentor and his large team. They never explicitly discussed how to do this, or what boundaries are optimal between their work. Carla has a small grant on how an individual's social media usage is related to treatment adherence in transplant recipients, and is thinking that she should forge relationships with a mathematician and machine learning expert to build a more sophisticated model and niche distinct from her mentor. But she is not quite sure how to do that. Moreover, Carla is nervous that any graduate students will choose to work with her old mentor rather than her. What steps would you advise her to do?

Reference 1: *The Nature and Evolution of the Mentoring Relationship in Academic Health Centers*

Reference 2: *Building and sustaining mentor interactions as a mentee*

TOPIC 11: Developing career-stage-specific cohorts of mentors

Case 1: Dr. Emmet Richter has is in his second year as an Assistant Professor of Hematology & Oncology. He left his institution for Mecca U that had a spot in their breast cancer program for a new faculty member. He is realizing that while his fellowship mentor was excellent in guiding his clinical training and research that culminated in his new K grant, he had received little advice about myriad tasks needed for transition to faculty. When he had submitted his K proposal, he had a mentoring team from his now former institution. There is not a formal onboarding program at Mecca U and he has struggled with everything from IRB submissions and animal protocols to tissue procurement and how to hire, who may be good collaborators, what the expectations and timeline are for his research productivity and whether he is answerable to the many ad hoc

whims of the clinical coordinator and his colleague who does the clinical schedule. He feels that his science and K grant plan is solid, but that is what his nominal mentor at Mecca U mostly wants to advise him on besides suggesting that he join the protocol review committee. Dr. Richter needs to find someone to guide him through logistics, help him know what is realistically expected of him both clinically and in research, be a sounding board as he thinks about hires and budgeting, help protect his time, and advocate for him. He is thinking this may be more than one person. How does he find them?

Case 2: Dr. Nona Bigood is an Associate Professor with substantial funding and a good reputation in her field. Many house staff and younger faculty come to her for advice or for mentoring. Often as her career advances, she feels like she is “winging it,” and this is becoming harder as she advances, with more leadership responsibilities and an elderly parent who is becoming demented and increasingly needs her. While she has friends that she vents to, she increasingly feels a need for a mentor to help her strategize about her responsibilities and path forward. But she is embarrassed to reach out, since she thinks that with her record she should be (or is viewed as) self-sufficient. How should she proceed?

- Reference 1: *Mapping a Mentoring Roadmap and Developing a Supportive Network for Strategic Career Advancement*
- Reference 2: *Caution Before Embracing Team Mentoring in Academic Medical Research Training: Recommendations from a Qualitative Study*
- Reference 3: *Selection of Research Mentors for K-Funded Scholars*
- Reference 4: *Intellectual synthesis in mentorship determines success in academic careers*

TOPIC 12: Maximizing collaborations and networking – promising or premonition

Case: You have a provocative finding that depression can be dramatically decreased by a protocol that includes training subjects to plan their dreams and to be conscious that they are dreaming. You think that if you can get collaborators on board to do EEGs or maybe fMRI scans that you can submit this work for a paper and a R01 grant. You approach several neuroscientists who seem only mildly interested and raise a number of caveats that you hadn't thought about. One of them though is interested enough so that if you provide money for the coordinator and EEG costs that they could do 10 subjects. You don't know though if that will be enough subjects to do more than show feasibility. You would like them to forego costs as much as possible since this is at such an early stage (and you only have a small amount of pilot support). How would you proceed? What are pros and cons of this situation?

- Reference 1: *Comprehensive Collaboration Plans: Practical Considerations Spanning Across Individual Collaborators to Institutional Supports*
- Reference 2: *How to pick a great scientific collaborator*

TOPIC 13: Research during post-graduate training—strategies to maximize productivity and benefit without duress

Case 1 (credit to Heather Acuff, MD, PhD): Sarah is a third-year anesthesiology resident. Throughout residency, she has been approached by multiple attendings who were excited by her MD/PhD background. These attendings asked if she wanted to work on multiple different kinds of projects. Eager to continue doing research, she initially said yes to working on every project that was offered. However, as she continued throughout residency, she realized that she had less time than she would have liked to work on research while also maintaining her clinical skills. Additionally, as she learned more about anesthesiology, her clinical interests changed, which also changed her research interests. How can she go about choosing which projects to

work on? How can she navigate difficult discussions with attendings? How can she manage her time between research and clinical responsibilities?

Case 2 (credit to Heather Acuff, MD, PhD): Jack is a second-year anesthesiology resident. Early on in residency, he focused on learning clinical skills and achieving work-life balance. He did not receive much guidance from his residency program regarding when to start doing research or how to go about finding a mentor. By the end of his second year of residency, he felt like he had achieved a good level of clinical competency, but he felt behind on his research. He tried to seek out research mentors during dedicated research months, but many of his attendings were busy and did not reach back out to him in timely manners, which further delayed his ability to start research. Who can he reach out to in order to obtain help in finding a mentor? When is the right time to start finding a research mentor in residency? How can he best make use of his dedicated research months throughout residency?

- Reference 1: *Overcoming the Obstacles to Research During Residency*
- Reference 2: *How to Succeed in Research During Fellowship: What the Trainee Needs To Do (and Needs From the Program)*

TOPIC 15: How to build your investigative team – deciding who and when to hire

Case: Dr. Jean James is a physician-scientist who has recently received funding that will enable him to hire either a second postdoc or a graduate student for his research on the role of immune cells in cancer progression. He receives many postdoc applications, and pulls several from individuals with extensive experience in immunology although no one has substantial experience in B cells and tertiary lymphoid structures, his area of investigation. On the other hand, there is a clearly intelligent and motivated incoming graduate student, Olivia Workman, who had spoken to Dr. James about her interest. However, her graduate coursework is heavy over the next year and a half and Dr. James has some concerns about her available effort for

lab work. He decides to Zoom interview 4 of the postdoc candidates. A current graduate student of his, knowing of the funding, asks “Is Olivia coming to the lab?” and praises her. Dr. James shares that he has not yet decided on who will fill the spot. How should he proceed? What should be deciding factors one way or the other?

- Reference 1: *For the "good of the lab": Insights from three focus groups concerning the ethics of managing a laboratory or research group*
- Reference 2: *How to build a motivated research group*
